# A de novo atypical ring sSMC(22) characterized by array CGH in a boy with cat-eye syndrome

**DOI:** 10.1186/1755-8166-7-37

**Published:** 2014-06-05

**Authors:** Irén Haltrich, Henriett Pikó, Eszter Kiss, Zsuzsa Tóth, Veronika Karcagi, György Fekete

**Affiliations:** 1 2nd Department of Paediatrics, Semmelweis University, Tűzoltó utca 7-9, Budapest 1094, Hungary; 2 Department of Molecular Genetics and Diagnostics, National Institute of Environmental Health, Budapest, Hungary

**Keywords:** Small supernumerary marker chromosome, sSMC, ring(22), Cat eye syndrome, Microarray

## Abstract

**Background:**

Microduplications 22q11 have been characterized as a genomic duplication syndrome mediated by nonallelic homologous recombination between region-specific low-copy repeats. Here we report on a 19 years old boy with intellectual disability having an unexpected structurally complex ring small supernumerary marker chromosome (sSMC) originated from a larger trisomy and a smaller tetrasomy of proximal 22q11 harboring additional copies of cat eye syndrome critical regions genes.

**Results:**

Principal clinical features were: anorectal and urogenital malformations, total anomalous pulmonary venous return with secundum ASD, hearing defect, preauricular pits, seizure and eczema. The proband also presented some rare or so far not reported clinical findings such as hyperinsulinaemia, severe immunodeficiency and grave cognitive deficits.

Chromosome analysis revealed a mosaic karyotype with the presence of a small ring-like marker in 60% of cells. Array CGH detected approximately an 1,2 Mb single and a 0,2 Mb double copy gain of the proximal long arm of chromosome 22. The 1,3 Mb intervening region of chromosome 22 from centromere to the breakpoints showed no copy alteration. The karyotype of the patient was defined as 47,XY,+mar[60]/46,XY[40].ish idic r(22)(q11.1.q11.21) × 4.arr 22q11(17,435, 645-18,656,678) × 3,(17,598,642-17,799,783) × 4 dn.

**Conclusions:**

The present report is the first one with a detailed description of clinical presentation in a patient carrying an atypical size ring sSMC (22) analyzed by array CGH. The specialty of the finding is emphasized by the fact that although the patient had a mosaic sSMC and the amplified region was smaller than in typical cat eye syndrome cases, the clinical presentation was severe.

## Background

The sSMC 22q has been characterized as a genomic duplication syndrome mediated by nonallelic homologous recombination between region-specific low-copy repeats, designated LCR22s (eight different, ranging from LCR22-A to LCR22-H) [1] or by der(22)t(11;22)(q23.3;q11.2) originated from malsegregation of a parental balanced translocation between chromosomes 11 and 22 (known as Emanuel Syndrome, OMIM #609029) [2]. A subset of SMCs is derived from proximal part of 22q11 and confers the tri- or tetrasomy for cat-eye syndrome (CES; OMIM #115470) chromosomal region. The original CESCR spans around 2Mb, from the centromere to the D22S57 marker, and includes two genes which are major candidates for relevant clinical features of CES: CECR1 between 17,659,680–17,702,744 Mb and CECR2 flanked by 17,956,630-18,033,845 Mb [3,4]. Interstitial duplication of CESCR has been also reported in patients with clinical picture of CES, but without an sSMC [5].

There are two typical CES chromosomes: the smaller symmetrical type I CES chromosomes do not contain the DiGeorge critical region because both breakpoints are located in the proximal interval of the 22q11 deletion/duplication syndrome. The larger type II CES chromosomes harbor one (asymmetrical) or two (symmetrical) distal breakpoints and enclose one or two copies of DiGeorge critical region [6]. Bartsch et al [7] described a third form (type III) bisatellited sSMC(22) with distal breakpoints outside of the LCRs22.

The more frequent attribute of CES phenotype [OMIM 115470] includes micrognathia, preauricular pits and/or tags, ocular coloboma, heart and kidney defects, genitourinary anomalies, anal atresia, normal to mild intellectual disability. The penetrance of these dysmorphic features is highly variable.

However over 210 cases of 22q11.2 microduplications have been reported so far, most of them overlap with the 3 Mb common typically deleted region (TDR) or 1,5 Mb DiGeorge critical region found in DGS/VCFS. The ring-like sSMCs generally occur with a lower frequency (0,14-0,72/100 in newborns) [8] and there are only a few details on ring sSMC(22) cases [9-11]. Here we characterize by array CGH an atypical ring sSMC with detailed description of clinical findings.

## Case presentation

**Figure 1:**
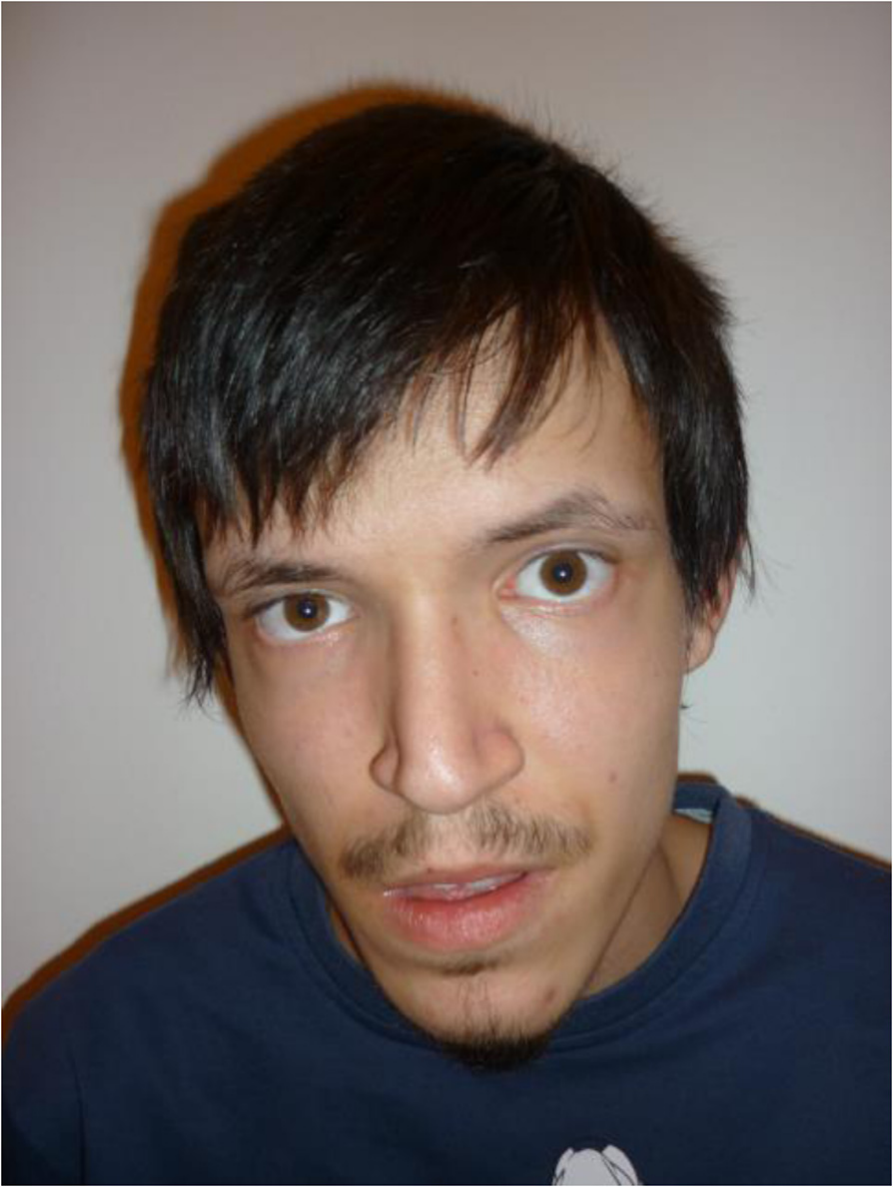
**Craniofacial features of the patient at the age of 17 years.** Note the long face, broad forehead, facial asymmetry, left microtia, downslanting palpebral fissures, long philtrum and thin upper lip.

The proband was born at the 36^th^ week of gestation as first child from non-consanguineous healthy parents. The proband’s BW was 2.500 g, BL 50 cm. At birth, he was diagnosed with total supracardiac anomalous pulmonary venous return with secondary atrial septal defect resolved by cardiac surgery. He needed also surgical intervention for inguinal hernia and later for varicocele. His craniofacial features included long face, broad forehead, facial asymmetry, left microtia, downslanting palpebral fissures, bilateral preauricular pits, long philtrum and thin upper lip (Figure 1). The ophthalmologic examination revealed Duane anomaly, myopia and strabismus. No fundal coloboma was noticed. The consequent bilateral conductive hearing loss was resolved by tympanostomy tube placement. Musculoskeletal abnormalities were the following: kypho-scoliosis, torticollis, longer fingers and toes, rich palm drawing and pes planus. The somatic developmental milestones were close to the normal range, he had moderate intellectual and severe language skills impairment. He could communicate in short phrases in hypernasal speech and he attended a school for the disabled. At the age of six years he presented neurogenic bladder with urinary incontinence. From the neonatal period to the present age the main health problems of the propositus were recurrent respiratory (otitis media, sinusitis, tonsillitis, mastoiditis usually associated with herpes and stomatitis) and urinary tract infections. He had long infection periods caused by Staphylococcus aureus with grave palm and plantar eczema. Because of severe hypogammaglobulinaemia at age of 2 years, he benefited from intravenous immunoglobulin substitution during two years. From his infancy he had complicated febrile seizures. At the age of 8 years EEG and MRI of the brain revealed frontal type epilepsy which needed combined antiepileptic treatment. His gastrointestinal symptoms (pyloric stenosis, malformation of the colon, gastroesophageal reflux, chronic colitis, permanent constipation and encopresis) needed surgical intervention several times and contiguous laxative and antacid medication. He has had hyperinsulinaemia and susceptibility to hypoglycaemia. Anxiety, mutism and behavioral changes appeared at the age of 16 years. Neuro-psychiatric examination revealed mixed disturbance of emotions and conduct, he is under medical and psychotherapeutical treatment from that time.

## Methods

Chromosomes for cytogenetic analysis were obtained from 72-hour lymphocyte cultures. Analysis of the sSMC, present in G-banded karyotype was carried out using whole chromosome painting (WCP) 22 and acrocentric p-arm probes (Cytocell Technologies, Ltd., Cambridge, UK), satellite enumeration probe, SE 14/22, DiGeorge (N25, N85A3), subtelomeric 22q and 11q (Vysis/Abbott, Inc., Downers Grove, IL, USA) and a 22q11.1q11.21 specific oligonucleotide probe spanning 17,427-18,040 Mb close to the centromere (Agilent SureFISH, Santa Clara, CA USA).

To ascertain the segmental composition of the sSMC, a high resolution genomic scan using ISCA plus design array of Nimblegen-Roche containing 1.4 M probes per subarray (assembly GRCh37/hg19) was performed in the DNA samples of the patient. This CGX microarray provides a mean average resolution of approximately 15-20 Kb. The subarrays were scanned on NimbleGen MS 200 microarray scanner and data were extracted and analysed using NimbleScan, SignalMap and Deva 1.1 softwares (Roche NimbleGen Inc.). DNA CNVs were mentioned as gain or loss in a linear ratio and the length of the variation was given in megabase (Mb).

## Results

**Figure 2:**
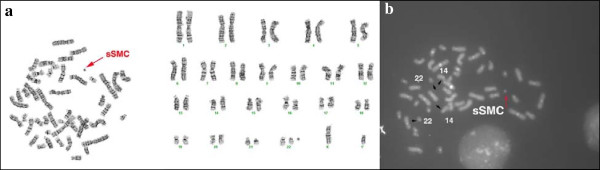
G-banding metaphase and karyotype (a) and DAPI-stained metaphase (b) with sSMC (red arrow) that appear ring-like.

**Figure 3:**
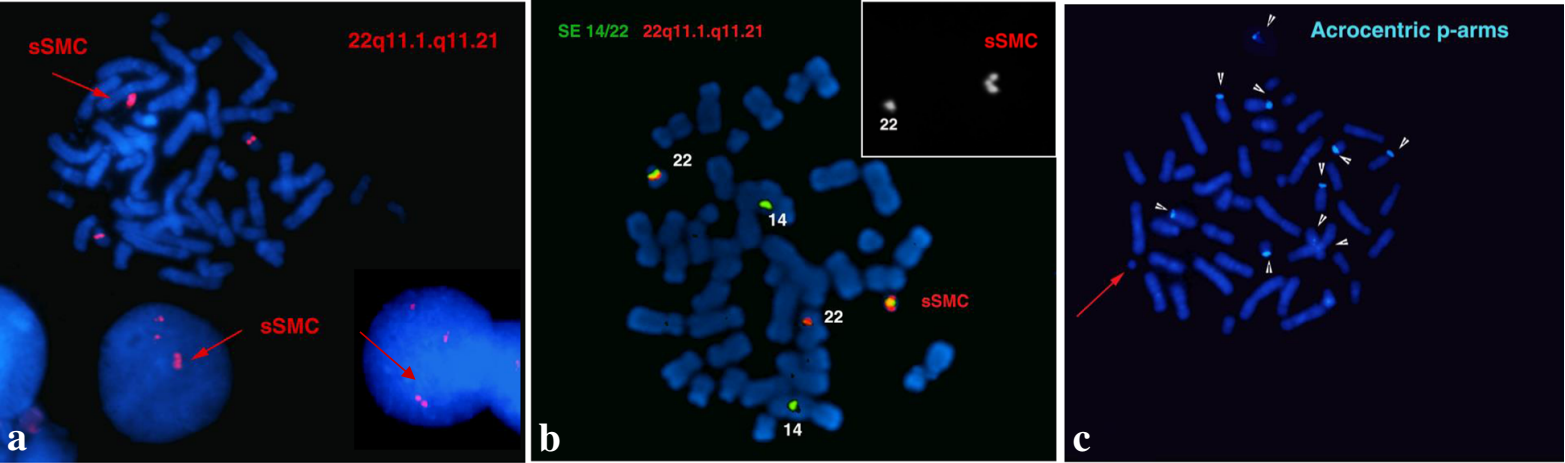
**The FISH result with CES critical locus.** The FISH analysis with 22q11 locus **(a)**, cohybridization with 22q11 red and 14/22 green centromere probes shows two normal hybridization 22q11 signals on homologues 22 and a duplicated on the sSMC(22) (red arrow). In the smaller box: enlarged red channel with 22q11 region FISH pattern of the normal chromosome 22 and sSMC(22) of the same metaphase showing the opposite localization to the centromere(s) of the two 22q duplicated segments **(b)**. The FISH with aqua acrocentric p-arm probe **(c)** shows signals on homologues 13, 14, 15, 21, 22 acrocentric chromosomes (white arrowhead), but this hybridization did not yield any signal on the round shaped sSMC(22) ( red arrow).

**Figure 4:**
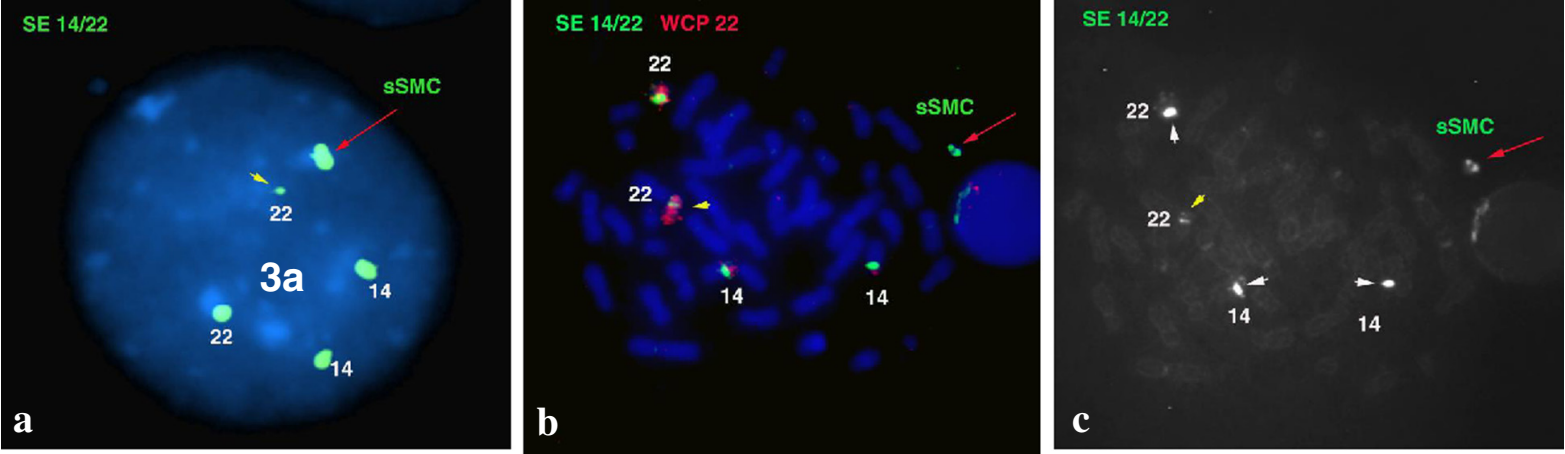
**The SE 14/22 green centromeres (a) SE 14/22 green centromeres and WCP 22 red probes FISH pattern of sSMC and normal homologous of 14/22 chromosomes (b), FITC channel (c) showing dicentric aspect of the sSMC 22 (red arrow) in interphase and metaphase cells.** Denote that one homologue 22 showed a very weak centromere signal (yellow arrow head).

Chromosome analysis revealed a mosaic karyotype with the presence of a small ring-like marker in 60% of cells in the patient (Figure 2a). FISH investigations using WCP 22 resulted two normal intensity chromosome 22 signals and a third very weak signal on sSMC. DAPI chanel showed a round shape of the sSMC (Figure 2b, see also on Figure 3c). Cohybridization with SE 14/22 green and WCP 22 red probes identified the normal diploid pattern of the chromosomes 14/22 and a large extra centromere on sSMC (Figure 4). Denote that one of the chromosome 22 showed a very weak centromere signal (yellow arrow head on Figure 4a, b, c) and the greater centromere of the sSMC had an adjacent, dicentric aspect in all analyzed metaphase and interphase cells (red arrow on Figure 4a, b, c). The patient’s mother presented also a very weak centromere signal for one of the homologous 22. The FISH probes for the DiGeorge critical region as well as the subtelomeric 22q and 11q presented normal diploid pattern for chromosome 22 and 11 and no signals on the sSMC.

**Figure 5:**
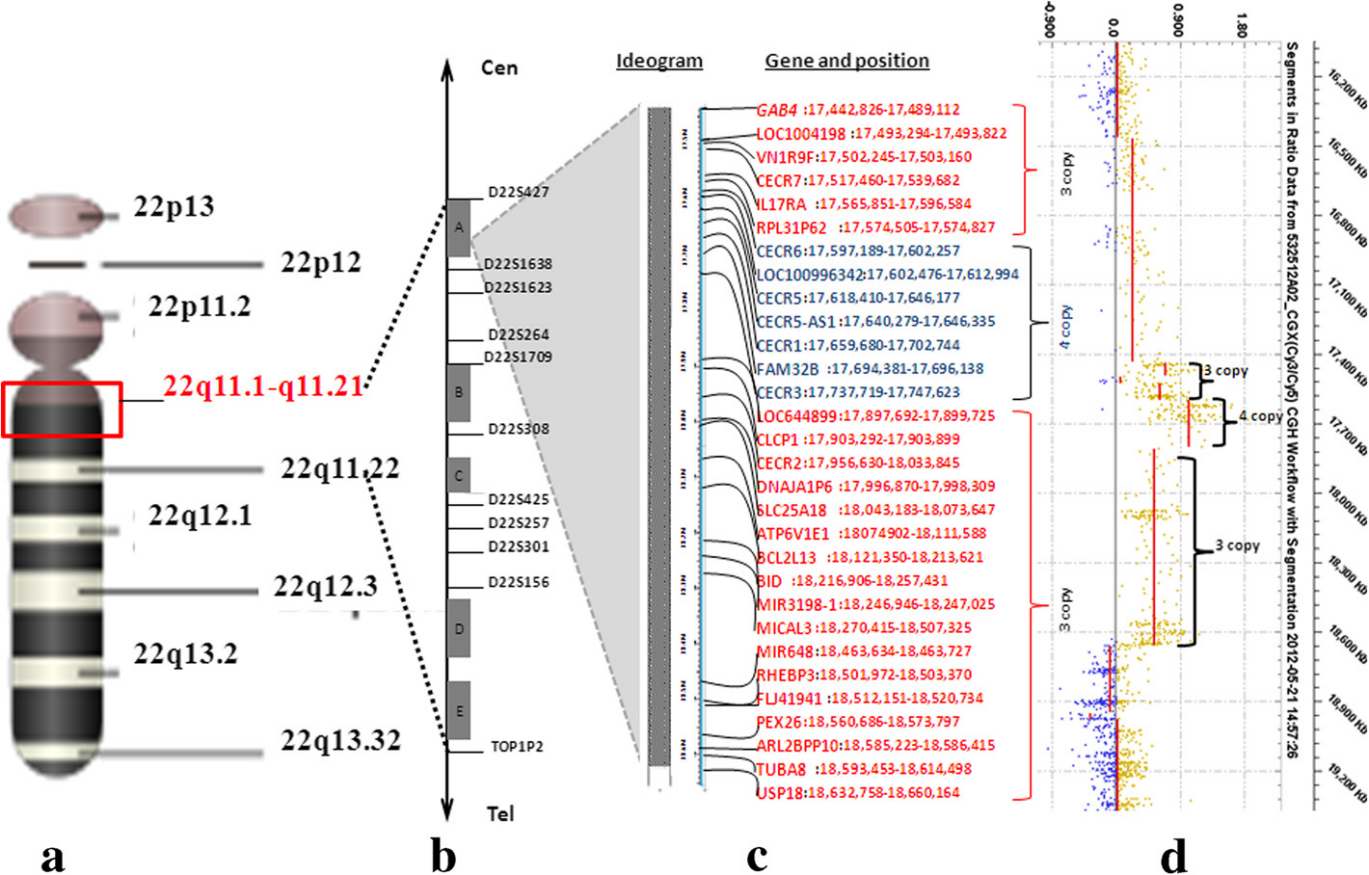
**Result of the array CGH genotyping (GRCh37/hg19) and mapping of the genomic gain, originating from chromosome 22.** Cytogenetic loci on chromosome 22 **(a)**. The red rectangle indicates the length of a 1.221 Mb size gain on chromosome 22q11.1-q11.21 in the male patient. Schematic representation of the chromosome 22q11.11-22q11.22 region with delineated genomic regions **(b)**. Gray boxes indicate Low copy repeat regions LCR22s (A-E). The arrow numbers represent the microsatellite markers (from D222S427 to TOP1P2), cen:centromere, tel: telomere. Panel **(c)** shows the enlarged LCR22 “A” region, which is affected in the patient. Next to the ideogram the affected genes and positions are listed, single copy gains are marked by red whereas double copy gains are marked with blue colour. Panel **(d)** reveals the result of the array CGH genotype. The CGX ISCA plus array (assembly GRCh37/hg19) showed a 1.221-Mb gain, which is separated in three regions: an 0.1629 Mb length region which has 3 copies (the red base line is above zero with around 0.5 log2 score), an 0.2011 Mb length region which has 4 copies (the red base line is above zero with around 1.0 log2 score) and an 0.8568Mb length region which has 3 copies (the red base line is above zero with around 0.5 log2 score).

**Figure 6:**
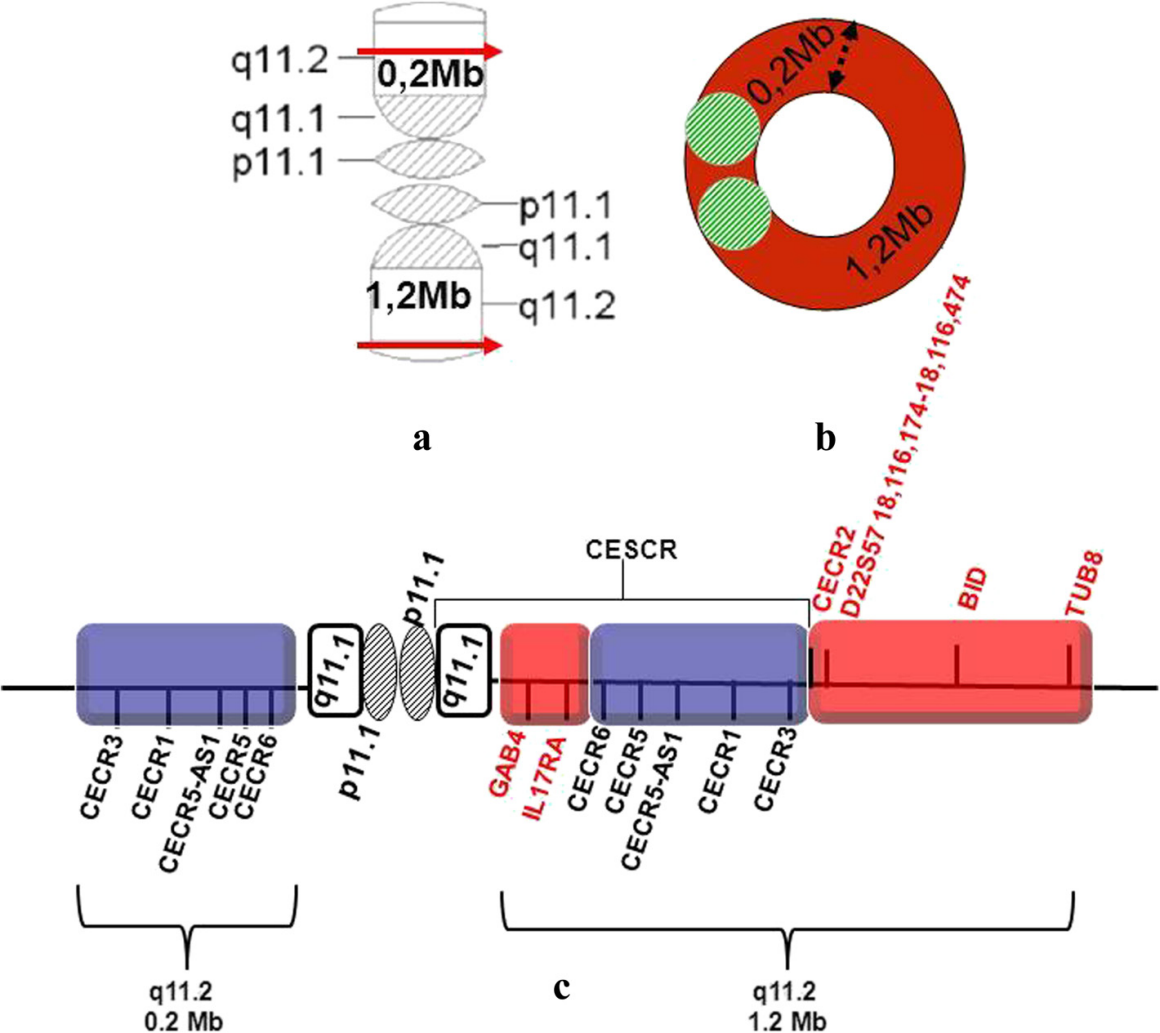
**Graphic representation of the proposed arrangement of the sSMC(22).** Schematic ideogram of sSMC(22): linear form of chromosome 22q segments situated vis-à-vis **(a)** and its ring derivative **(b)**. The red arrows indicating the breakpoints, the black dashed arrow the fusion points, gray and green lined circle the centromeres. Diagram of the amplified 22q 11 interval with most relevant genes **(c)**. Blue segments represent double copy and red segments single copy gains.

Array CGH detected approximately an 1,2 Mb single and a 0,2 Mb double copy gain of the proximal long arm of chromosome 22. The 1,3 Mb intervening region of chromosome 22 from centromere to the breakpoints using 313 probes with 4,4 kb coverage, showed no copy alteration (Figure 5). Subsequently, FISH was performed using a 22q11.1q11.21 oligonucleotide probe close to the centromere. This encompassed both copy gain segments (spanning 17,427-18,040 Mb) and confirmed the array finding exhibiting double signals on the marker chromosome (Figure 3a). Co-hybridization with this confirming red 22q11 oligonucleotide and green centromere 14/22 showed that the two 22q11 signals of sSMC were located in an opposite position (vis à vis), distal to the centromere (Figure 3b, see also enlarged red channel of 22q11 probe). The hybridization of the acrocentric p-arms probe identified the acrocentric choromosomes 13, 14, 15, 21, 22 and did not yield any signal on the sSMC in 50 marker carrier metaphases (Figure 3c) indicating that the short arm of chromosome 22 was lacking from the sSMC. These findings suggested that the presented sSMC was very likely dicentric (Figure 4), composed from two different sizes of proximal part of the 22q arm: one 1,2 Mb large one comprehending 17,435,645-18,656,678 Mb and another one smaller (0,2 Mb) flanking 17,598,642 to 17,799,783 Mb (Figure 5d, Figure 6). The opposite localization to the centromere(s) of the two 22q duplicated segments (Figure 3b) and the differently stained cytogenetic aspects of the marker (Figures 2a,b, 3c) suggested that this sSMC(22) was a ring chromosome. On the basis of these results the karyotype of the patient was defined as 47,XY,+mar[60]/46,XY[40].ish idic r(22)(q11.1.q11.21) × 4.arr 22q11(17,435,645-18,656,678) × 3,(17,598,642-17,799,783) × 4 dn. Cytogenetic analysis of both parents demonstrated normal karyotypes and regular chromosomes 22 FISH patterns in lymphocyte and buccal cells, indicating that the sSMC(22) had arisen de novo.

## Discussion

Classical CES is usually associated with a supernumerary isodicentric bisatellited chromosome 22 symmetrical or asymmetrical, depending on the proximal or distal localization of the two recurrent deletion/duplication breakpoints [9].

We identified an asymmetric dicentric sSMC(22) originated from a larger trisomy and a smaller tetrasomy of 22q11 harboring two additional copies of CECR1 and one of CECR2, and no extra copies of DiGeorge critical regions and chromosome 22 satellites. The ring shape was suggested by G-band and DAPI stained aspect of sSMC. The symmetric localization of the two 22q11 control oligonucleotid segments on the FISH images led us to hypothesize that the starting point of this sSMC(22) was a classical inv dup(22)(q11.21) dicentric chromosome and its breakages would have led to an “inv dup del” that stabilizes itself through circularization. All of the proximal and distal duplication breakpoints of the ring sSMC stretched into the largest, LCR22-A region which is considered to be the recurrent proximal deletion endpoint of TDR seen in 85% of 22q11.2 deletion syndrome cases (Figure 5a, b) [1]. However, similar breakpoints and even the same gene content have been published [12,13], were not specifically in ring shape. The reported ring 22 chromosomes were accompanied by the absence of normal chromosome and/or were the results of centric fission 22 [11,14-16]. The majority of ring sSMC(22) cases was prenatally detected, with only some poor clinical details, lacking a precise delineation by sensible techniques such as microarray [9,10]. The present report is the first one with a detailed description of clinical presentation in a patient carrying a ring sSMC(22) atypical in size which was analyzed by array CGH. Eventhough, the presented patient’s CES chromosome was in 60% mosaic and smaller than typical type I CES, he carried almost all major (cardiovascular, urogenital and gastro-intestinal) and minor (micrognathia, preauricular pits, kypho-scoliosis, pes planus) as well neurological (speech delay, intellectual disability) abnormalities characteristic for CES. He suffered from some medical issues commonly reported in individuals with Emanuel syndrome such as recurrent urinary tract and ear infections, sinusitis, low immunoglobulin levels, constipation, gastroesophageal reflux, hearing loss [2]. The proband additionally presented some rare or previously not reported clinical findings such as hypoglycaemia, hyperinsulinaemia and psychiatric troubles.

The gene content of the present sSMC(22) (Figures 5c,d, 6c) included two extra copies of *CECR1*(OMIM 607575), *CECR3* and *CECR6* and a single additional copy of *CECR2* (OMIM 607575), *CECR7 CESCR* candidate genes. Homo sapiens *CECR1* gene encodes two distinct adenosine deaminases, ADA1 and ADA2. The ADA1 deficiency causes severe combined immunodeficiency syndrome and ADA2 protein may act as a growth factor and has adenosine deaminase activity converting adenosine and deoxyadenosine to inosine and deoxyinosine, respectively. Extra-cellular adenosine is an important regulatory molecule with a low physiological concentration that can rapidly increase during tissue damage and inflammation. ADA2 is specifically secreted by antigen-presenting cells and may navigate to sites with a high concentration of adenosine by binding to specific cell surface receptors [17]. The role of ADA2 is to induce differentiation of monocytes into macrophages as well as to stimulate the proliferation of macrophages and CD4+ T cells. The presented patient suffered from severe immunodeficiency, recurrent episodes of bacterial infection with sepsis, almost permanent herpes and/or stomatitis. We can speculate that ADA2 overhead production due to the tetrasomy of CECR1 could perturb the role of extra-cellular adenosine in adaptive immunity [18]. Current discovered high resolution protein structure of ADA2 revealed two specific protein domains of novel folds that mediate the protein dimerization and are responsible for the receptor-mediated growth factor activity of ADGF/ADA2 proteins [17]. 

*CECR1* gene expression in the outflow tract and atrium of the heart as well as in the facial/auditory-vestibular cranial nerve ganglion suggests its involvement in heart malformation and facial defects. Recent studies have been demonstrated that extra-cellular adenosine can act as an anti-insulin hormone, stimulating a release of glucose from stores in the Drosophila model [19]. In this model increased extracellular adenosine is associated with hyperglycemia and impairment in energy storage. A similar role for adenosine signaling through adenosine receptors stimulating, the glucose release was described for mammalian liver cells [20], suggesting that this role of extracellular adenosine and the mechanism of action are evolutionary conserved from flies to mammals.

The GAB4 is a member of docking proteins family closely related to the insulin growth factor, cytokine and antigen receptors as well as cell adhesion molecules contributing to signal diversification by channeling the information from activated receptors into signaling pathways. These scaffold proteins play critical roles in protein-protein interactions in a variety of physiological processes as well as in disorders including cancer and inflammation, Alzheimer and cardiovascular diseases [21,22]. We can hypothesize that gene dosage alterations of GAB4 and ADA2 and their possible functional interactions could be subject of changes in carbohydrate metabolism and these are likely the cause of hypoglycaemia susceptibility of the presented patient. It is noteworthy that transient hypoglicaemia similar to those seen in our patient have also been reported in a previous CES phenotype r(22) case [16].

The *CECR2* is known as a chromatin remodeling gene, its candidate downstream genes are mesenchymal and ectodermal transcription factors involved in neural tube closure and inner ear development [23,24]. Overexpression influence on the development of brain, eye and ear might be responsible for frequent abnormalities of these organs in CES patients. In our patient, the most frequent eye abnormality, coloboma was absent; instead he had Duane anomaly described only in a smaller number of CES patients [9].

*MICAL3* (OMIM 608882) and *TUBA8* (OMIM 605742) are responsible for cytoskeletal structures essential for neuronal migration and function [25], *BID* (OMIM 601997) regulates the cross-talk of cell cycle arrest and apoptosis [26]. The overdosage of these three distal genes could be related to the neurologic impairment in our as well as in other CES patients.

The protein encoded by *IL17RA* (interleukin 17A receptor, OMIM 605461) gene is a ubiquitous type I membrane glycoprotein that binds with low affinity to interleukin 17A. Interleukin 17A and its receptor play a pathogenic role in many inflammatory and autoimmune diseases. Increased gene dosage could negatively impact the CES patient’s infectious conditions. The altered immune response function of this gene is not mentioned in other CES reports [27].

Out of the thirteen patients with ring sSMC(22) listed in the sSMC database [9] not a single one was identical with the case presented here and only two cases (reported by Ballif et al [28] and SW Cheung with scarce clinical description) were analyzed by array CGH. A similar but smaller double ring sSMC(22) was described by Mears et al in 1995 [4]. The fact that both patients (published by Mears and presented here) were carriers of a smaller than typical type I CES with four copies of *CECR1* and possessed all main CES abnormalities suggests that *CECR1* gene dosage and functional interactions among amplified genes [12] are the predictable determining factor to express the CES phenotype and the size of sSMC(22) is rather a contributing factor to phenotypic variability.

## Conclusions

Here we fully characterized a highly complex ring sSMC(22) containing extra copies of CES critical regions by high resolution array CGH. On basis of the sSMC database our ring is a unique finding being atypical in size and construction. The proband presented so far rare or not reported clinical findings such as hypoglycaemia, hyperinsulinaemia, severe immunodeficiency and grave cognitive deficits. The array CGH provided to be an optimal technique to determine the duplication breakpoints while chromosome banding provided information on mosaic feature and ring morphology of the sSMC. The FISH examination with whole chromosome painting probes is not a sufficiently sensitive technique to identify the origin of sSMCs but the use of adequate oligonucleotide probes is an efficient tool for confirmation of the array finding and level of mosaicism. This case illustrates the importance of reporting rare and unusual CES chromosome carrier patients to delineate more precisely the genotype-phenotype correlations and to evaluate the incidence of this type of ring sSMC(22) in the general population.

## Consent

Written informed consent was obtained from the patient’s parents for publication of this case report, portrait and accompanying images. A copy of the written consent is available by the Editor-in –chief of this journal.

## Abbreviations

ADA1 and ADA2: Adenosine deaminases; ASD: Atrial septal defect; CES: Cat-eye syndrome; CESCR: CES critical region; CGH: Comparative genomic hybridisation; GAB4: GRB2-Associated binding protein family, member 4; IL17RA: (interleukin 17A receptor) gene; LCR22s: Low-copy repeats of chromosome 22; MICAL3: Microtubule associated onoxygenase, calponin and LIM domain containing 3 gene; SE: Satellite enumeration probe; sSMC: Small supernumerary marker chromosome; TDR: Typically deleted region; TUBA8: Tubulin, alpha 8 gene; BID BH3: Interacting domain death agonist isoform 1; WCP: Whole chromosome painting probe.

## Competing interest

The authors declare that they have no competing interests.

## Authors’ contributions

GyF and IH cared for the patient and his family. IH contributed to data collection and the first draft of the manuscript. HP and VK performed the array CGH analysis. ZsT and IH performed cytogenetic examination, EK and IH performed FISH examination. VK and GyF read and approved the final manuscript. All authors read and approved the final manuscript.
